# Supplementary material for: Transcriptional programs regulating neuronal differentiation are disrupted in DLG2 knockout human embryonic stem cells and enriched for schizophrenia and related disorders risk variants
Source: Nat Commun. 2022 Jan 14;13:27. doi: 10.1038/s41467-021-27601-0 (PMC8760302; doi:10.1038/s41467-021-27601-0)
Supplement: Supplementary file 1 — Supplementary Information [file 41467_2021_27601_MOESM1_ESM.pdf]

Supplementary Information

Supplementary Figure 1
Supplementary Figure 2
Supplementary Figure 3
Supplementary Figure 4
Supplementary Figure 5
Supplementary Figure 6
Supplementary Figure 7
Supplementary Figure 8
Supplementary Figure 9

Supplementary Data 1
Supplementary Data 2
Supplementary Data 3
Supplementary Data 4
Supplementary Data 5
Supplementary Data 6
Supplementary Data 7
Supplementary Data 8
Supplementary Data 9
Supplementary Data 10

Supplementary Software

Supplementary Software file contains scripts for RNA sequencing data analysis and downstream bioinformatic/human genetic analyses, plus files containing annotated gene sets. Further details can be found in enclosed README files. For access to human genetic data please see Data availability statement.

* Supplementary Data (excel file) and Supplementary Software (zip file) are attached separately.

a 1st gRNA sequence - 5’ GGCTTCCACCGCTTTACTGT 3’
2nd gRNA sequence - 5’ AGGAAGCAGGGTCTATCGTT 3’

b Forward primer: 5’ ATCTTGCGGGTGAATGAGGTTG
Reverse primer: 5’ GTCGTCTTCTACGCACATACAG

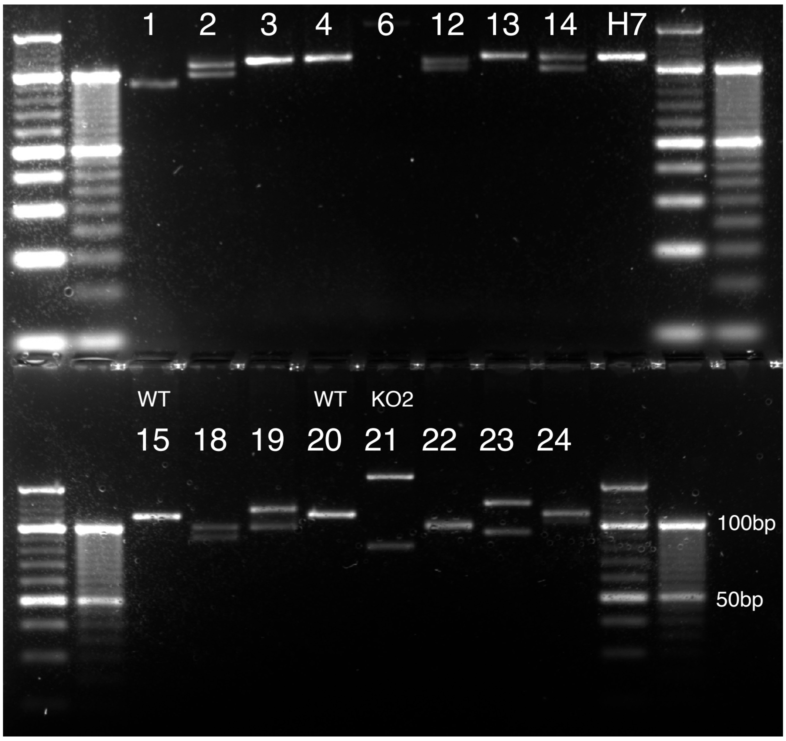

c

Cut by Cas9 nickase

WT 5′GAGGTTGATGTGTCAGAGGTTTCCCACAGTAAAGCGGTGGAAGCCCTGAAGGAAGCAGGGTCTATCGTTCGG
CTC CAACTACACAGTCTCCAAAGGGTGTCATTTCGCCACCT TCGGGACT TCCT TCGTCCCAGATAGCAAGCC5′

Cut by Cas9 nickase

KO1 allele 1 5′GAGGTTGATGTGTCAGAGGTTTCCCACAGTAAAGCG~~GTGG~~AAGCCCTGAAGGAAGCAGGGTCTATCGTTCGG

KO1 allele 2 5′GAGGTTGATGTGTCAGAGGTTTCCCACAGTAAAGC~~GGTGGAAGCCCTG~~AAGGAAGCAGGGTCTATCGTTCGG

KO2 allele 1 5′GAGGTTGATGTGTGGGAAACCTCTGAACAGGATTATCTGAGGTTCTCCTTGAACTTTGTAAATCTGAACTTCAG
TATTTATAATATTATAAGATGAATCGAGGTGGAG~~CAGAGGTTTCCCACAGTAAAGCGGTGGAAGCCCTGAAGGA~~
AGCAGGGTCTATCGTTCGG

KO2 allele 2 5′GAGGTTGATGTGTCAGAGGTTTCCCACAG~~TAAAGCGGTGGAAGCCCTGAAGGAAGCAG~~GGTCTATCGTTCGG

d

WT	V D V S E V S H S K A V E A L K E A G S I V R L Y V R R R R P I L E T V V E I K L F K G P K G L G
KO1 allele 1	V D V S E V S H S K A K P *
KO1 allele 2	V D V S E V S H S K A R K Q G L S F G C M C V E D D L F W R P L W K S N C S K A L K V *
KO2 allele 1	V D V W E T S E Q D Y L R F S L N F V N L N F S I Y N I I R *
KO2 allele 2	V D V S E V S H R V Y R S A V C A *

Supplementary Figure 1. Generation of *DLG2*$^{-/-}$ human embryonic stem cell lines
a, Two guide RNA sequences used to target exon 22 of *DLG2* which is translated into part of the 1st PDZ domain. **b**, A primer set used for screening successful indels on targeted DNA. PCR and gel electrophresis shows several hESC clones with indels which were confirmed in three independent experiments. Parental H7 lines shows a single 109bp band whereas several clones, such as 2 and 21, show two different sized bands suggest- ing generation of indels on the targted exon. **c**, PCR amplicons were sequenced and clones no.2 and 21 found to be homozygous knockouts (named KO1 and KO2). Red letters indicate deleted bases while blue indicate inserted DNA. **d**, Indels in KOs generated premature stop codons on the targeted exon. Green indicates different amino acid sequences from WT.

53 .. 97 Export to GENBANK

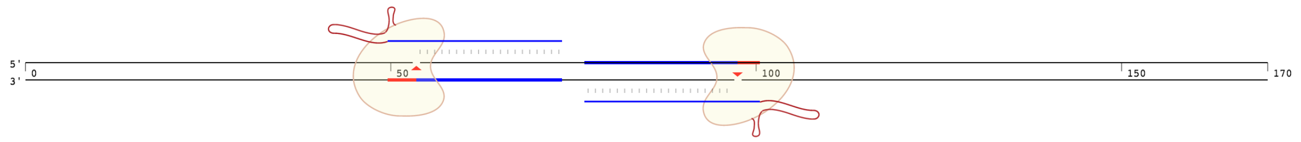

Overhang region

GGTCAATGATTGTATCTTGCGGG
TGAATGAGGTTGATGTGTCAGAG
GTTTCCCACAGTAAAGCGGTGGA
AGCCCTGAAGGAAGCAGGGTCTA
TCGTTCGGCTGTATGTGCGTAGA
AGACGACCTATTTTGGAGACCGT
TGTGGAAATCAAACTGTTCAAAG
GCCCTAAAG

Guide A

81

quality
high
cuts after position
53 in query
sequence
GGCTTCCACCGCTTTACTGTGGG
# offtargets
89
# genic offtargets
18
Export off-targets to .csv

Guide B

90

quality
high
cuts after position
97 in query
sequence
AGGAAGCAGGGTCTATCGTTCGG
# offtargets
125
# genic offtargets
14
Export off-targets to .csv

Pair score, A & B

74

quality
high
# offtarget pairs of A & B
0
# genic OT pairs of A & B
0

Supplementary Figure 2. Quality of two gRNAs used in the study
Seperately the gRNAs had prediced off-targets of 89 and 125 loci each; however, the predicted off-targets when used as a pair was none. Captured from crispr.mit.edu.

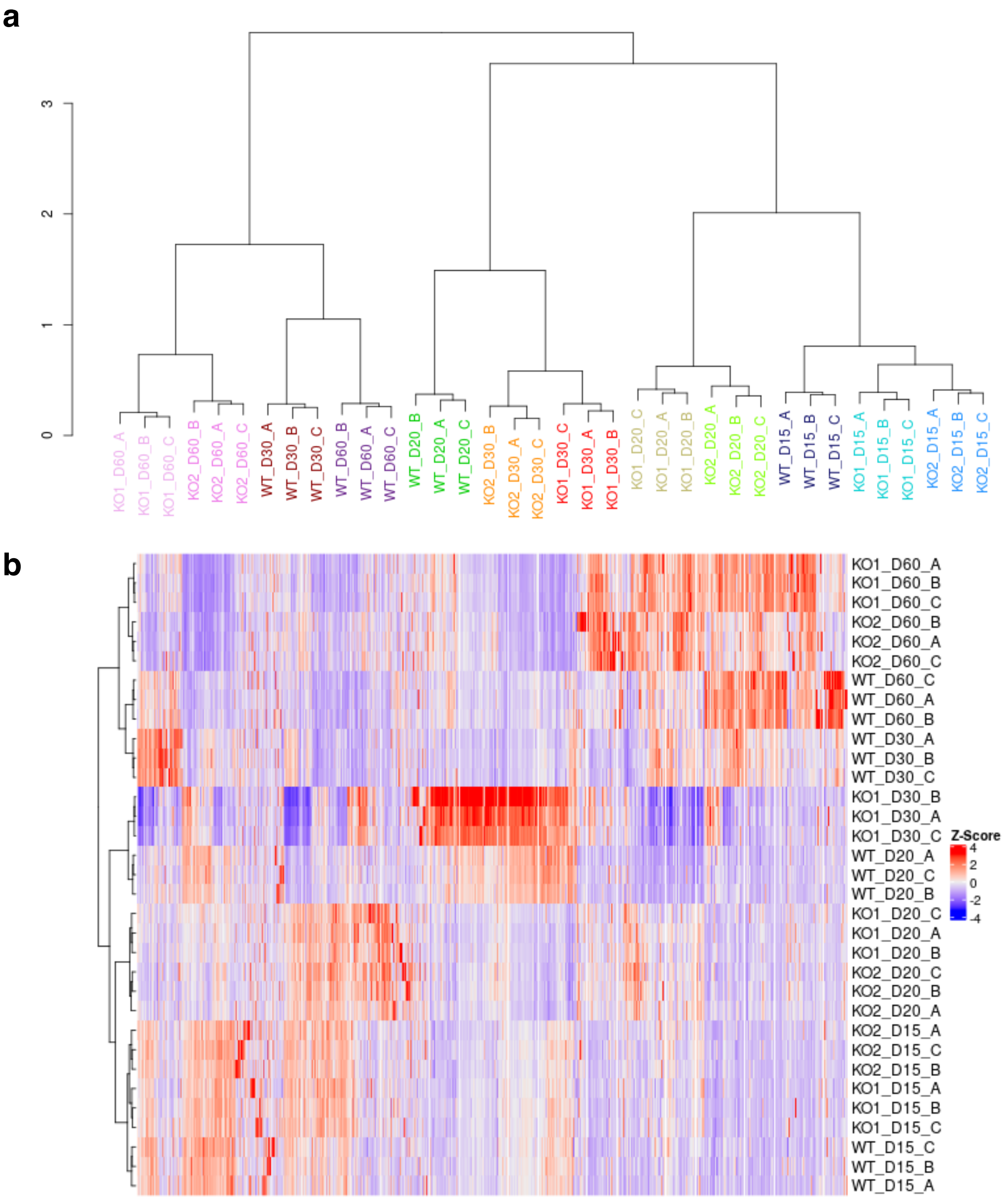

Supplementary Figure 3. Clustering and visualisation of KO and WT sample gene expression. **a,** Hierarchical clustering of KO and WT samples based on relative expression of protein-coding genes (20,004 genes). Clustering was performed on a Pearson's correlation matrix of the z-scored gene expression, using Ward's linkage criterion (R hclust option '*ward.D2*') and 1-r as the distance function, where r is the pairwise Pearson's correlation coefficient. **b,** Heatmap showing transcriptomic profiles of KO and WT samples (low-quality KO2 samples from day 30 removed) based on the z-scored expression levels of variably expressed protein-coding genes (coefficient of variation > 25%; 15,003 genes). Heatmap clustering was performed as in **a**, using Pearson's correlation matrix and Ward's linkage criterion. Clustering and heatmap both illustrate the similarity between KO lines and their delayed development relative to WT.

a

Day 30

Start	End	Domain	Exon ID	KO	WT	log_2fold-KO-WT	P	$P_{corrected}$
84059484	84059435	PDZ1	ENSE00003484205	0.023	4.60	-15.29	0.0018	0.0016
84098994	84098923	PDZ1	ENSE00003586389	0.023	4.54	-15.26	0.0020	0.0019

Day 60

Start	End	Domain	Exon ID	KO	WT	log_2fold-KO-WT	P	$P_{corrected}$
83965468	83965324	PDZ2	ENSE00003483868	7.64	13.05	-1.56	$6.38x10^{-17}$	$1.03x10^{-14}$
83980642	83980561	PDZ3	ENSE00003595386	6.17	10.62	-1.57	$1.02x10^{-12}$	$9.56x10^{-11}$
84059434	84059315	PDZ2	ENSE00003484205	4.89	10.47	-2.21	$2.66x10^{-12}$	$2.34x10^{-10}$
83980560	83980506	PDZ1	ENSE00003595386	6.28	10.57	-1.51	$9.00x10^{-12}$	$7.26x10^{-10}$
83963023	83962963	PDZ2~3	ENSE00003607494	6.43	11.10	-1.58	$6.63x10^{-10}$	$3.96x10^{-8}$
84059484	84059435	PDZ1	ENSE00003484205	4.63	8.79	-1.85	$1.42x10^{-9}$	$8.05x10^{-8}$
84098994	84098923	PDZ1	ENSE00003586389	5.15	8.81	-1.56	$3.47x10^{-7}$	$1.23x10^{-5}$
83786792	83786690	PDZ3	ENSE00003542004	6.61	8.83	-0.84	0.00041	0.0062
83962962	83962885	PDZ2~3	ENSE00003607494	6.51	11.20	-1.57	0.00048	0.0071
84163511	84163461	PDZ1	ENSE00003616457	4.41	8.05	-1.74	0.0012	0.015
83833770	83833614	PDZ3	ENSE00003544320	6.48	8.32	-0.73	0.0022	0.024

b

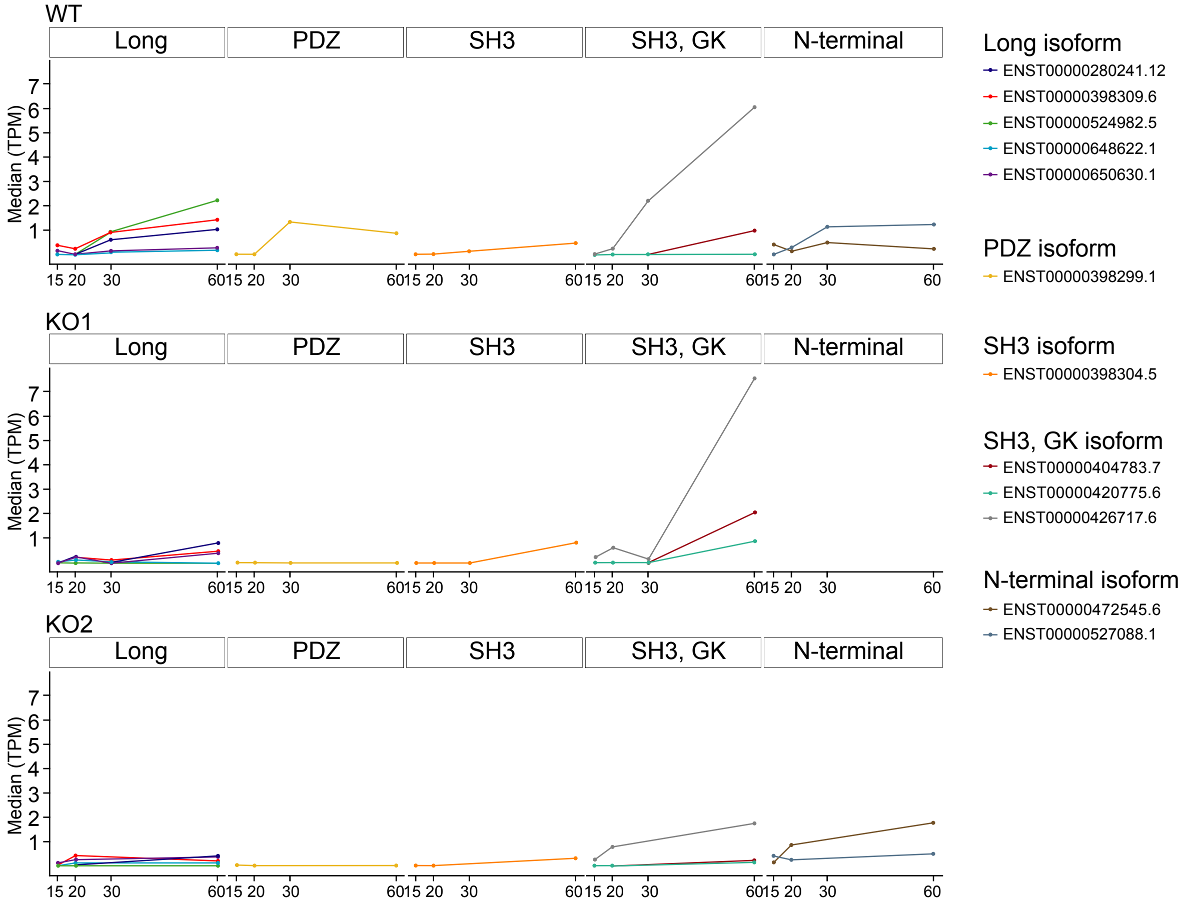

Supplementary Figure 4. Validation of *DLG2*$^{-/-}$ human embryonic stem cells

a, Differential exon usage (days 30 and 60) for exons encoding *DLG2* PDZ domains. Analysis was performed using DEXseq. Shown are all exonic regions significant at $P_{corrected}$ < 0.05 following FRD correction for multiple testing. **b**, *DLG2* predicted transcript expression in WT, KO1 and KO2 lines at each time point. Transcript-level counts in TPM (Transcripts Per Million) were imported from RSEM using tximport (v1.12.3) (Soneson et al., 2015). Shown are all protein-coding transcripts consistently identified (TPM > 0 in all replicates) in at least one time point. Transcripts were classified according to the functional domains they contain and the median TPM plotted for each time point. Long transcripts contain all 3PDZs, SH3 and GK domains.

a

Cell line	CNV location	Duplication/Deletions	Position	Length
WT, KO1, KO2	2q37.3	Deletion	chr2:242900000-243100000	200Kb
WT, KO1, KO2	3q26.1	Duplication	chr3:166000000-1664000000	400Kb
WT, KO1, KO2	20p12-p11.1	Deletion	chr20:91000-25889300	25.8Mb
WT, KO1, KO2	20q11.21-q13.33	Duplication	chr20:26088528-62707975	36.6Mb

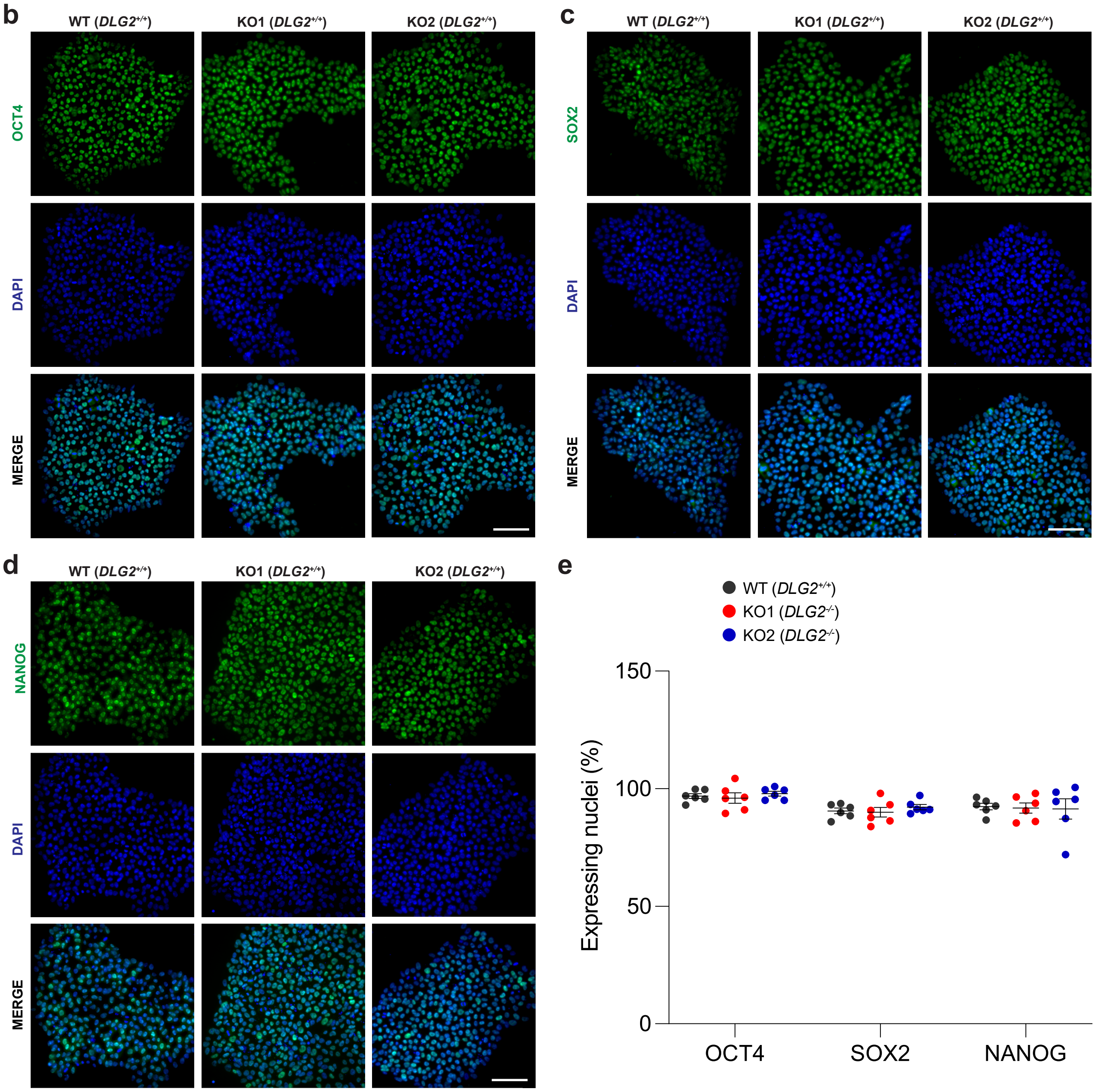

Supplementary Figure 5. Quality control of *DLG2*$^{-/-}$ human embryonic stem cells

a, Copy number variant analysis of *DLG2*$^{-/-}$ hESCs. WT, KO1 and KO2 genomic DNAs were analysed using Illumina PsychArray v1.1 and data analysed using PennCNV. CNVs smaller than 100Kb and containing less than 10 SNPs were filtered out in the PennCNV QC. No additional CNVs are identified in both KO lines in comparison to WT line. **b**-**e**, Pluripotency marker expression of *DLG2*$^{-/-}$ hESCs. Key pluripotency transcription factors were expressed both in 2 *DLG2*$^{-/-}$ hESC lines and WT controls. Representative ICC images of OCT4 (**b**), SOX2 (**c**) and NANOG (**d**) expression with DAPI nuclear counterstain for 2 *DLG2*$^{-/-}$ hESC lines and WT controls. **e**, ICC quantification of nuclei expressing either OCT4, SOX2 or NANOG in 2 *DLG2*$^{-/-}$ hESC lines and WT controls, the genotype had no significant effect on the expression of these markers (OCT4: $F_{2,15}$=0.3780, P=0.9616, n=6; SOX2: $F_{2,15}$=0.5383, P=0.5946, n=6; NANOG: $F_{2,15}$=0.03433, P=0.9663, n=6). Analysis was by one-way ANOVA for each pluripotency marker. All data presented as mean ± SEM and all scale bars are 100 μm.

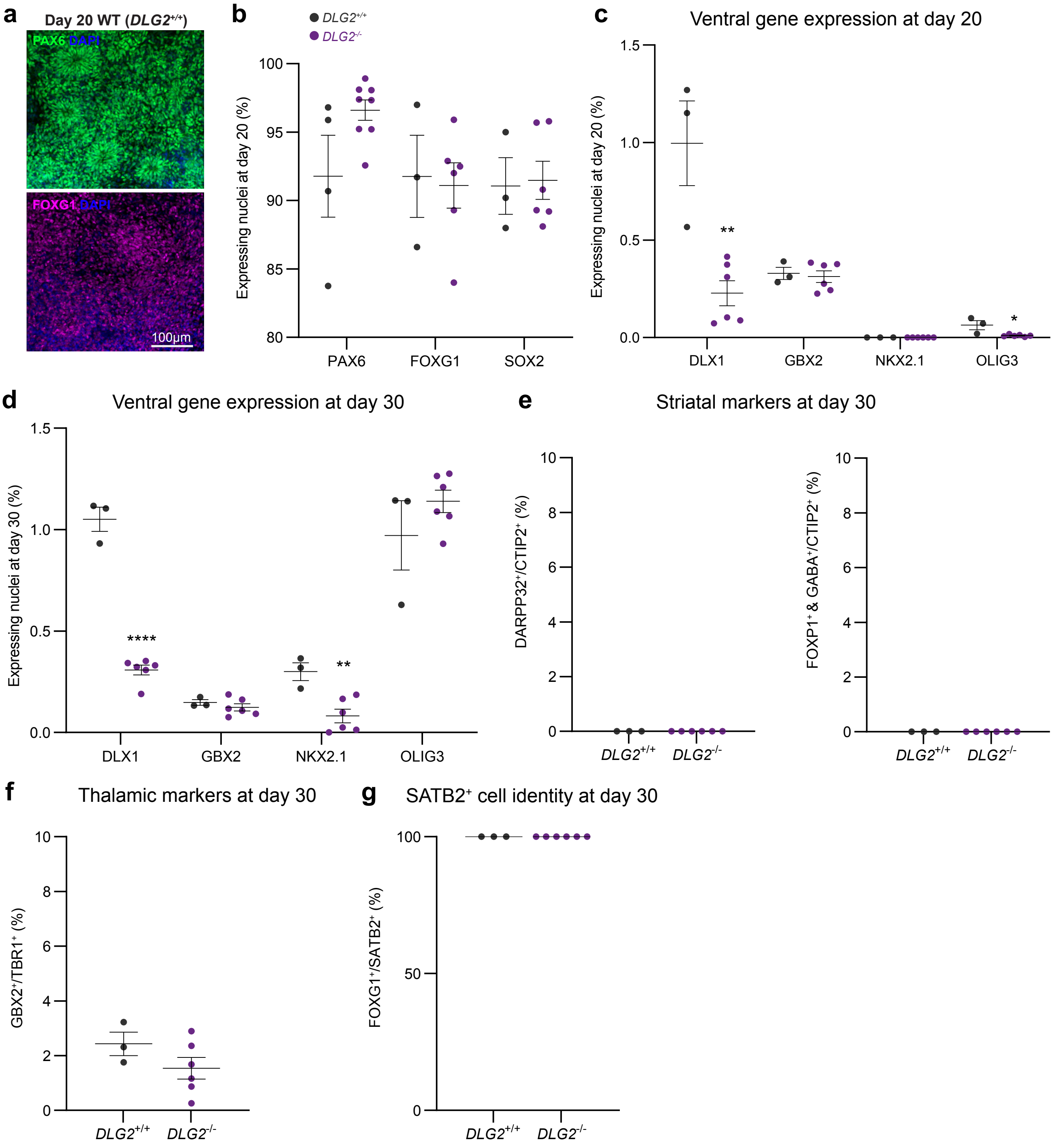

Supplementary Figure 6. Cell fate characterisation of WT and *DLG2*$^{-/-}$ cells during cortical differentiation

a, Representative ICC images showing expression of forebrain progenitor markers PAX6 and FOXG1 at day 20 of cortical differentiation. **b**, ICC quantification of nuclei expressing either PAX6, FOXG1 or SOX2 in *DLG2*$^{-/-}$ and WT cells at day 20 of cortical differentiation, the genotype had no significant effect on the expression of these markers (PAX6: t_{10}=2.113, P=0.0607, n=4, 8 for WT and KO; FOXG1: t_7=0.2133, P=0.8372, n=3, 6 for WT and KO; SOX2: t_7=0.1702, P=0.8697, n=3, 6 for WT and KO). **c**, ICC quantification of nuclei expressing either DLX1, GBX2, NKX2.1 or OLIG3 in *DLG2*$^{-/-}$ and WT cells at day 20 of cortical differentiation. Cells expressing these markers were minor, less than 1%. n= 4, 6 for WT and KO for each staining. DLX1: t_7=4.521, P=0.0027; GBX2: t_7=0.3386, P=0.7448; OLIG3: t_7=3.414, P=0.0112. **d**, ICC quantification of nuclei expressing either DLX1, GBX2, NKX2.1 or OLIG3 in *DLG2*$^{-/-}$ and WT cells at day 30 of cortical differentiation. Cells expressing these markers were minor. n= 4, 6 for WT and KO for each staining. DLX1: t_7=14.05, P=2.19x10^{-6}; GBX2: t_7=0.8917, P=0.4021; NKX2.1: t_7=3.875, P=0.0061; OLIG3: t_7=1.219, P=0.2623. **e**, None of the CTIP2$^+$ cells co-expressed striatal markers such as DARPP32 or FOXP1 and GABA. n=3, 6 for WT and KO. **f**, Minority of TBR1$^+$ cells expressed the thalamic marker GBX2. t_7=1.379, P=0.2105, n=3, 6 for WT and KO. **g**, All SATB2$^+$ cells expressed the telencephalic marker FOXG1. n=3, 6 for WT and KO. All data set except for **e** and **g** was analysed using unpaired two-tailed Student's t-tests for each marker investigated. All data presented as mean ± SEM where possible. *, p<0.05; **, p< 0.01; ****, p<0.0001 compared to WT.

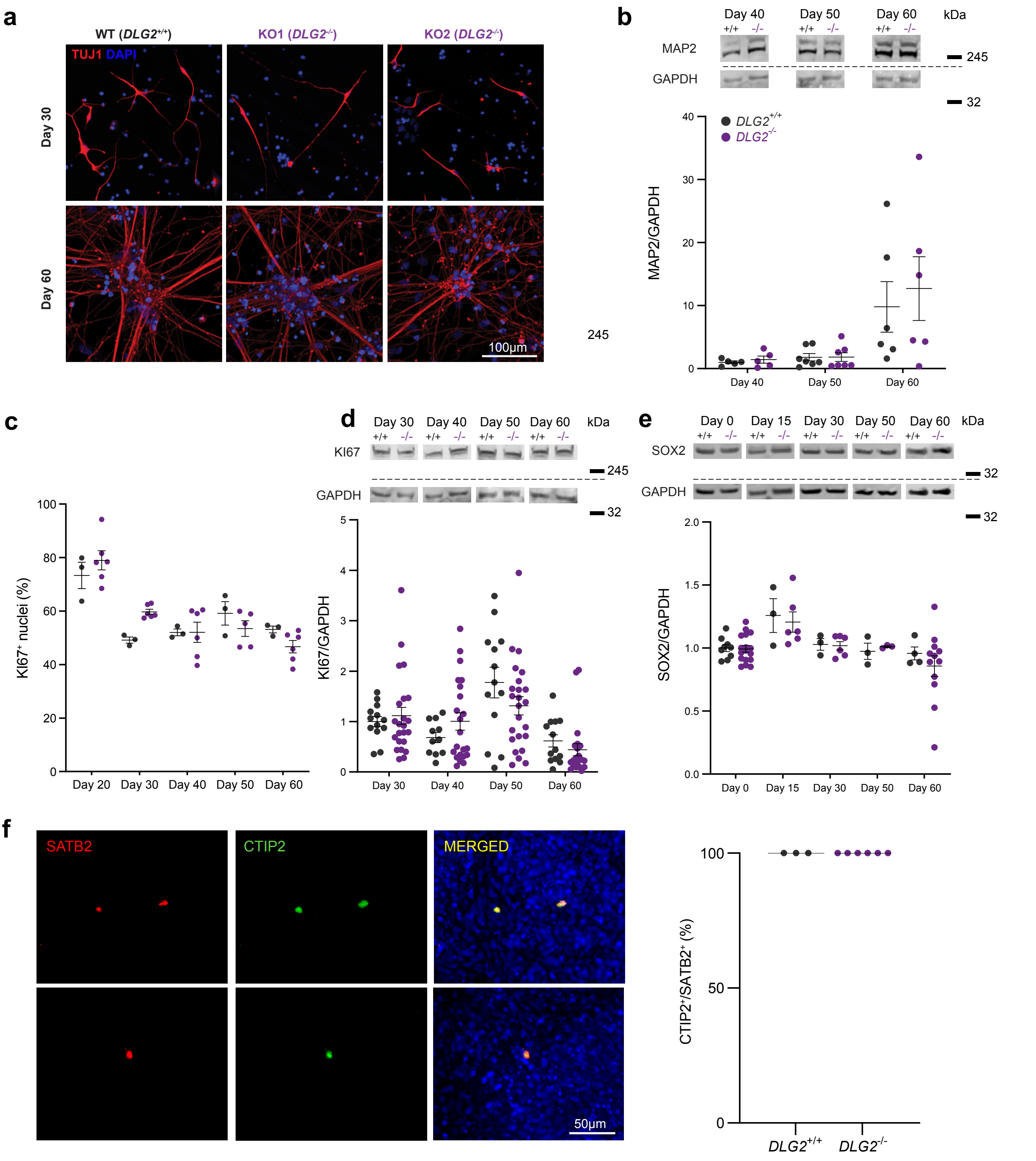

Supplementary Figure 7. Further characterisation of WT and *DLG2*$^{-/-}$ cells during cortical differentiation **a**, Representative ICC images showing the expression of the neuronal marker TUJ1 (β-Tubulin III) in 2 *DLG2*$^{-/-}$ lines and WT controls at days 30 and 60 of cortical differentiation. **b**, MAP2 western blot protein bands and histograms of expression normalised to GAPDH and day 40 WT expression for *DLG2*$^{-/-}$ and WT cells at 3 time points of cortical differentiation. n=5, 7, 6 from day 40 to 60 for WT and KO. Time ($F_{2,30}$=8.721, P=0.0010) did have a significant effect on MAP2 expression, while genotype ($F_{1,30}$=0.2673, P=0.6157) did not. **c**, ICC quantification of KI67 expressing nuclei for *DLG2*$^{-/-}$ and WT cells at 5 time points of cortical differentiation. n=3 for WT at all time points, n=6 for KO at all timepoints except n=5 at day 50. Time ($F_{4,34}$=20.71, P=1.00x10^{-8}) did have a significant effect on KI67 expression, while genotype ($F_{1,34}$=0.1535, P=0.6976) did not. **d**, KI67 western blot protein bands and histograms of expression normalised to GAPDH and day 30 WT expression for *DLG2*$^{-/-}$ and WT cells at 4 time points of cortical differentiation. n=13, 11, 13, 13 for WT and n=23, 22, 24, 21 for KO from day 30 to 60. Time ($F_{3,132}$=10.94, P=1.83x10^{-6}) did have a significant effect on MAP2 expression, while genotype ($F_{1,132}$=0.1536, P=0.6957) did not. **e**, SOX2 western blot protein bands and histograms of expression normalised to GAPDH and day 0 WT expression for *DLG2*$^{-/-}$ and WT cells at 5 time points of cortical differentiation. n=9, 3, 3, 3, 4 for WT and n=17, 6, 6, 3, 12 for KO from day 0 to 60. Time ($F_{4,56}$=4.856, P=0.0020) did have a significant effect on SOX2 expression, while genotype ($F_{1,56}$=0.2928, P=0.5906) did not. **f**, Examples of SATB2^{+} cells co-expressing CTIP and quantification. n=3, 6 for WT and KO. All data sets except those in f were analysed by two-way ANOVA with post hoc comparisons using Bonferroni correction, comparing to WT controls. All data presented as mean ± SEM.

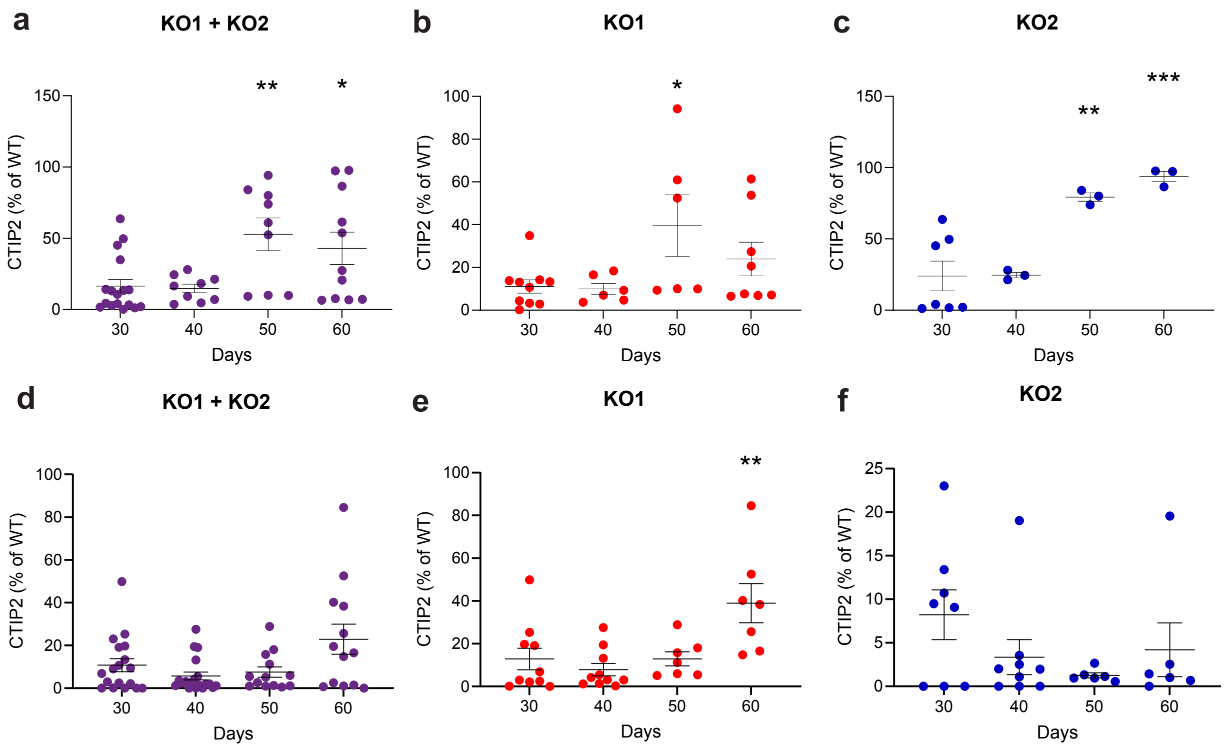

Supplementary Figure 8. CTIP2 analysis of two *DLG2*$^{-/-}$ cell lines

a, The proportion of CTIP2^{+} cells in both *DLG2*$^{-/-}$ lines relative to WT level from ICC analysis. One-way ANOVA $F_{3,42}$=5.391, P=0.0031, n=17, 9, 9, 11 from day 30 to 60. **b**, The proportion of CTIP2^{+} cells in KO1 *DLG2*$^{-/-}$ line relative to WT level from ICC analysis. One-way ANOVA $F_{3,26}$=3.053, P=0.0462, n=10, 6, 6, 8 from day 30 to 60. **c**, The proportion of CTIP2^{+} cells in KO2 *DLG2*$^{-/-}$ line relative to WT level from ICC analysis. One-way ANOVA $F_{3,12}$=12.61, P=0.0005, n=7, 3, 3, 3 from day 30 to 60. **d**, The level of CTIP2 protein expression in both *DLG2*$^{-/-}$ lines relative to WT level from Western blot analysis. One-way ANOVA $F_{3,59}$=3.939, P=0.0125, n=18, 19, 13, 13 from day 30 to 60. **e**, The level of CTIP2 protein expression in KO1 *DLG2*$^{-/-}$ line relative to WT level from Western blot analysis. One-way ANOVA $F_{3,30}$=6.375, P=0.0018, n=10, 10, 7, 7 from day 30 to 60. **f**, The level of CTIP2 protein expression in KO2 *DLG2*$^{-/-}$ line relative to WT level from Western blot analysis. One-way ANOVA $F_{3,25}$=1.493, P=0.2407, n=8, 9, 6, 6 from day 30 to 60. Data sets were analysed by one-way ANOVA, with post hoc comparisons using Bonferroni correction in all cases. Stars above bars represent Bonferroni-corrected post hoc tests, *P<0.05; **P<0.01; ***P<0.001 vs. day 30**.** All data presented as mean ± SEM.

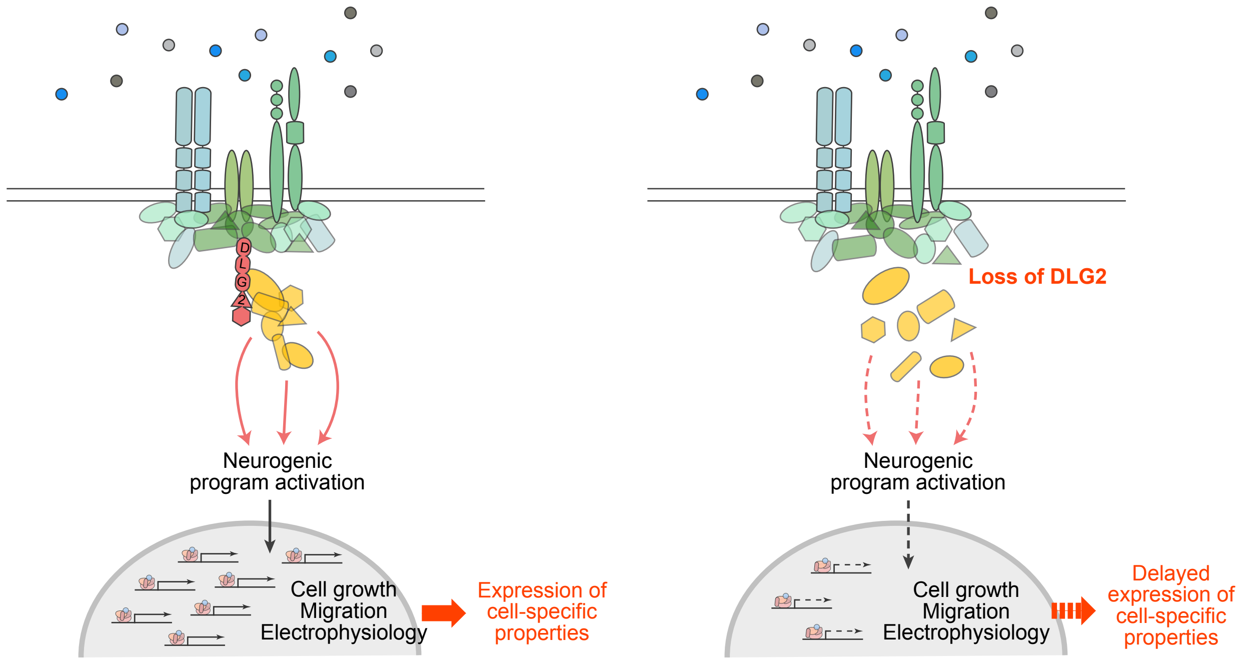

Supplementary Figure 9. Proposed model for DLG2 action in neurodevelopment

External cues transduced by DLG2-scaffolded complexes regulate transcriptional accessibility and/or activation of neurogenic programs underlying cell growth, migration and development of electrophysiological signalling properties. DLG2 knockout impairs signal transduction, disrupting the orchestration of events required for normal development and leading to stochastic, imprecise signaling that delays expression of cell-specific properties.
