# Supplementary material for: Transcriptional programs regulating neuronal differentiation are disrupted in DLG2 knockout human embryonic stem cells and enriched for schizophrenia and related disorders risk variants
Source: Nat Commun. 2022 Jan 14;13:27. doi: 10.1038/s41467-021-27601-0 (PMC8760302; doi:10.1038/s41467-021-27601-0)
Supplement: Supplementary file 3 — Description of Additional Supplementary Files [file 41467_2021_27601_MOESM3_ESM.docx]

**Description of Additional Supplementary Files**

Title: Supplementary Data 1.

Description: CRISPR/Cas9 off-target validation

Title: Supplementary Data 2.

Description: DLG2 unique peptides (LC-MS/MS of day 30 and 60 samples)

Title: Supplementary Data 3.

Description: Differential gene expression (KO v WT and successive WT timepoints)

Title: Supplementary Data 4.

Description: SZ association conditioning on combined/timepoint-specific expressed genes

Title: Supplementary Data 5.

Description: GO over-representation analysis (KO vs WT day 30 down-regulated genes)

Title: Supplementary Data 6.

Description: SZ association conditioning on *in vivo* foetal cortical neuron expressed genes

Title: Supplementary Data 7.

Description: GO over-representation analysis (neurogenic transcriptional programs)

Title: Supplementary Data 8.

Description: Schizophrenia GWAS enrichment (GO terms over-represented in early-stable^-/-^)

Title: Supplementary Data 9.

Description: GO term over-representation analysis of SZ-associated neurogenic program pyramidal^high^ gene-sets

Title: Supplementary Data 10.

Description: Expression of neurogenic programs across human *in vivo* neurodevelopmental cell-types

Title: Supplementary Software

Description: Supplementary Software file contains scripts for RNA sequencing data analysis and downstream bioinformatic/human genetic analyses, plus files containing annotated gene sets. Further details can be found in enclosed README files. For access to human genetic data please see Data availability statement.
