# Supplementary material for: Transcriptional programs regulating neuronal differentiation are disrupted in DLG2 knockout human embryonic stem cells and enriched for schizophrenia and related disorders risk variants
Source: Nat Commun. 2022 Jan 14;13:27. doi: 10.1038/s41467-021-27601-0 (PMC8760302; doi:10.1038/s41467-021-27601-0)
Supplement: Supplementary file 15 — Reporting Summary [file 41467_2021_27601_MOESM15_ESM.pdf]

nature research

Corresponding author(s): Eunju Shin

Last updated by author(s): Nov 26, 2021

Reporting Summary

Nature Research wishes to improve the reproducibility of the work that we publish. This form provides structure for consistency and transparency in reporting. For further information on Nature Research policies, see our Editorial Policies and the Editorial Policy Checklist.

Statistics

For all statistical analyses, confirm that the following items are present in the figure legend, table legend, main text, or Methods section.

n/a	Confirmed	
☐	☒	The exact sample size (*n*) for each experimental group/condition, given as a discrete number and unit of measurement
☐	☒	A statement on whether measurements were taken from distinct samples or whether the same sample was measured repeatedly
☐	☒	The statistical test(s) used AND whether they are one- or two-sided<br>*Only common tests should be described solely by name; describe more complex techniques in the Methods section.*
☐	☒	A description of all covariates tested
☐	☒	A description of any assumptions or corrections, such as tests of normality and adjustment for multiple comparisons
☐	☒	A full description of the statistical parameters including central tendency (e.g. means) or other basic estimates (e.g. regression coefficient) AND variation (e.g. standard deviation) or associated estimates of uncertainty (e.g. confidence intervals)
☐	☒	For null hypothesis testing, the test statistic (e.g. *F*, *t*, *r*) with confidence intervals, effect sizes, degrees of freedom and *P* value noted<br>*Give P values as exact values whenever suitable.*
☒	☐	For Bayesian analysis, information on the choice of priors and Markov chain Monte Carlo settings
☐	☒	For hierarchical and complex designs, identification of the appropriate level for tests and full reporting of outcomes
☐	☒	Estimates of effect sizes (e.g. Cohen's *d*, Pearson's *r*), indicating how they were calculated

Our web collection on statistics for biologists contains articles on many of the points above.

Software and code

Policy information about availability of computer code

Data collection	Zen 2012 SP2 (black) v11.02.190 (Zeiss), LAS X for DMI6000B inverted microscope (Leica), HCS studio Cell Analysis Software v6.6.0 (Thermo Fisher), IncuCyte software (Essen bioscience), Multiclamp 700B commander (Molecular Devices), Image Studio Lite Version 5.2 for Odyssey CLx (LI-COR), Tune Plus for Orbitrap Elite (Thermo Fisher), Genome Studio v2.0 software (Illumina)
Data analysis	R v3.6.1, FastQC v0.11.8 (Andrews, 2010), Cutadapt v2.3 (Martin, 2011), STAR v2.7.0e (Dobin et al., 2013), Samtools v1.9 (Li et al., 2009), Picard tools (v2.20.2), RSEM v1.3.1 (Li and Dewey, 2011), DESeq2 v1.24.0 (Love et al., 2014), DEXSeq v1.30 (Anders et al., 2012), HTSEQ v0.11.2 (Anders et al., 2015), Long-RNA-Seq pipeline (PsychENCODE Consortium), Variant Effect Predictor (McLaren et al., 2016), MAGMA v1.07 (de Leeuw et al. 2015), HCS Studio Cell Analysis Software v6.6.0 (Thermo Fisher), Prism v8.3.0 (Graph Pad), MaxQuant v1.6.10.43 (Cox and Mann, 2008), Genome Studio v2.0 (Illumina), PennCNV (Wang et al., 2007), WinLTP (Anderson and Collingridge, 2007), Clampfit v10.6 and 10.7 (Molecular Devices), SigmaPlot v12.5 (Systat Software), ImageJ (Schneider et al., 2012), StackReg plugin (Thevenaz, 2011), Neurolucida 360 v2.00.2 (MBF Bioscience), Image Studio Lite Version 5.2 for Odyssey CLx (LI-COR), Genome Studio v2.0 software (Illumina).<br>R scripts used to perform analyses are available in Supplementary Software.

For manuscripts utilizing custom algorithms or software that are central to the research but not yet described in published literature, software must be made available to editors and reviewers. We strongly encourage code deposition in a community repository (e.g. GitHub). See the Nature Research guidelines for submitting code & software for further information.

Data

Policy information about availability of data

All manuscripts must include a data availability statement. This statement should provide the following information, where applicable:

- Accession codes, unique identifiers, or web links for publicly available datasets
- A list of figures that have associated raw data
- A description of any restrictions on data availability

RNAseq data generated by this study have been deposited in the Gene Expression Omnibus (GEO) archive with accession number GSE172199 (https://www.ncbi.nlm.nih.gov/geo/query/acc.cgi?acc=GSE172199). The mass spectrometry data generated in this study have been deposited in the ProteomeXchange Consortium via the PRIDE partner repository with the dataset identifier PXD029526 (http://www.ebi.ac.uk/pride/archive/projects/PXD029526). Human proteome reference sequences are available in UniProt (https://www.uniprot.org/). Processed gene annotation data (GO terms, LoFi genes, BCL11B, TBR1, FMRP, TCF4, CHD8 target genes and Pyramidalhigh set) are available in the accompanying Supplementary Software zip file. The human foetal single cell RNAseq data used in this study are available in the UCSC Cell Browser repository (bit.ly/cortexSingleCell). GWAS data used in this study are available in the Psychiatric Genomics Consortium download database (https://www.med.unc.edu/pgc/download-results/) under accession codes: 29483656 (SZ, https://figshare.com/articles/dataset/scz2018clozuk/14681220), 31043756 (BP, https://figshare.com/articles/dataset/bip2019/14671998), 30478444 (ADHD, https://figshare.com/articles/dataset/adhd2019/14671965), 30804558 (ASD, https://figshare.com/articles/dataset/asd2019/14671989). The MDD GWAS data are available under restricted access due to inclusion of data from the Hyde et al. cohort (23andMe, Inc.), for which permission must be obtained separately. Access can be obtained by qualified researchers under an agreement with 23andMe that protects the privacy of the 23andMe participants. Contact David Hinds (dhinds@23andme.com) to apply for access to the data. Summary statistics for the MDD GWAS excluding the 23andMe data are available from the Psychiatric Genomics Consortium download database (https://www.med.unc.edu/pgc/download-results/) under accession code 29700475 (https://figshare.com/articles/dataset/mdd2018/14672085). The IQ GWAS data used in this study are available from (https://ctg.cncr.nl/software/summary_statistics/) as a compressed file (SavageJansen_IntMeta_sumstats.zip). The AD GWAS data used in this study are available from the authors of the original study (https://www.nature.com/articles/ng.2802). The SZ de novo rare variant data used in this study are available from the cited study (https://www.nature.com/articles/s41593-019-0565-2). The ASD and NDD de novo rare variant data used in this study are available in Supplementary Tables 1 and 4 of the cited study (https://www.cell.com/cms/10.1016/j.cell.2019.12.036/attachment/44aca411-6be3-4158-b1d6-6b339d60136d/mmc1.xlsx, https://www.cell.com/cms/10.1016/j.cell.2019.12.036/attachment/45f1baed-a2ec-4128-be82-a50ae08adc38/mmc4.xlsx). The GRCh38.p12 genome reference sequence and gene annotation data used for the bulk RNAseq analysis are available from GENCODE (https://ftp.ebi.ac.uk/pub/databases/gencode/Gencode_human/release_31/gencode.v31.primary_assembly.annotation.gtf.gz, https://ftp.ebi.ac.uk/pub/databases/gencode/Gencode_human/release_31/GRCh38.primary_assembly.genome.fa.gz).

Field-specific reporting

Please select the one below that is the best fit for your research. If you are not sure, read the appropriate sections before making your selection.

[x] Life sciences [ ] Behavioural & social sciences [ ] Ecological, evolutionary & environmental sciences

For a reference copy of the document with all sections, see nature.com/documents/nr-reporting-summary-flat.pdf

Life sciences study design

All studies must disclose on these points even when the disclosure is negative.

Sample size	RNA sequencing was performed on triplicate samples (conventional size) for each cell line at each time point. PCA analysis of these samples showed substantial differences between KO and WT cell lines and minimal distances between triplicates within the same cell line, demonstrating sufficient sample numbers. Proteomics was performed on quadruplicate samples (conventional size) for each cell line at each time point analysed to show the absence of DLG2 protein. In all KO samples, no DLG2 protein is detected with statistical significance compared to WT, demonstrating sufficient sample numbers.<br>Each phenotypic assay also followed conventional sample size and the results demonstrate sufficient sample numbers.
Data exclusions	A high level of duplicate reads in day 30 KO2 samples (~72% compared to an average of 23% for other samples) was found in quality assessment of RNAseq data using Picard tools. These samples were removed prior to further analyses.
Replication	All phenotypic assays were performed in a minimum of two independent differentiations. Within a given differentiation triplicate samples were used per cell line at each time point investigated.
Randomization	Assignment of WT and two KO hESC lines to the group cannot be performed in a random fashion. However, for the rest of the experiments randomization was implemented.
Blinding	Researchers were blinded to the genotype of cell lines except for RNAseq and Proteomics analysis. Those analysis was performed using objective methods so the knowledge of sample identity did not affect the results.

Reporting for specific materials, systems and methods

We require information from authors about some types of materials, experimental systems and methods used in many studies. Here, indicate whether each material, system or method listed is relevant to your study. If you are not sure if a list item applies to your research, read the appropriate section before selecting a response.

Materials & experimental systems

n/a	Involved in the study
	☒ Antibodies
	☒ Eukaryotic cell lines
☒	Palaeontology and archaeology
	☒ Animals and other organisms
☒	Human research participants
☒	Clinical data
☒	Dual use research of concern

Methods

n/a	Involved in the study
☒	ChIP-seq
☒	Flow cytometry
☒	MRI-based neuroimaging

Antibodies

Antibodies used

All antibodies used in the study are specified with the sources, identifiers, dilutions and applications in methods.
Rabbit polyclonal anti-Calnexin (Abcam ab22595), Rat monoclonal anti-CTIP2[25B6] (Abcam, ab18465), Rabbit polyclonal anti-CTIP2 (Abcam ab70453), Rabbit monoclonal anti-DARPP32 [EP721Y] (Abcam ab40802), Mouse polyclonal anti-DLX1 (Abcam ab167575), Rabbit polyclonal anti-FOXG1 (Abcam ab18259), Mouse monoclonal anti-FOXP1 [JC12] (Abcam ab32010), Rabbit polyclonal anti-GABA (Sigma A2052), Rabbit polyclonal anti-GAPDH (Abcam ab9485), Goat polyclonal anti-GBX2 (Antibodies.com A84236), Mouse monoclonal anti-KI67 [B56] (BD Pharmingen 550609), Mouse monoclonal anti-MAP2 [AP-20] (Merck Millipore AB5622), Rabbit monoclonal anti-NANOG [D73G4] (Cell Signaling Technology 4903), Rabbit polyclonal anti-NEUN (Merck Millipore ABN78), Rabbit monoclonal anti-NKX2.1 [EP1584Y] (Abcam ab76013), Rabbit monoclonal anti-OCT4 [C30A3] (Cell Signaling Technology 2840), Mouse monoclonal anti-OLIG3 [257934] (R&D Systems MAB2456), Rabbit monoclonal anti-PAX6 [EPR15858] (Abcam ab195045), Rabbit polyclonal anti-PSD-95 (DLG4) (Abcam ab18258), Mouse monoclonal anti-SATB2 [SATBA4B10] (Abcam ab51502), Rabbit monoclonal anti-SOX2 [D6D9] (Cell Signaling Technology 3579), Goat polyclonal anti-SOX2 (Santa Cruz sc-17320), Rabbit polyclonal anti-TBR1 (Abcam ab31940), Mouse monoclonal anti-β-Tubulin III (TUJ1) [SDL.3D10] (Sigma-Aldrich T8660), Donkey anti-Rabbit IgG (H+L) secondary antibody Alexa Fluor 488 (Thermo Fisher, A-21206), Donkey anti-Rat IgG (H+L) secondary antibody Alexa Fluor 488 (Thermo Fisher A-21208), Donkey anti-Mouse IgG (H+L) secondary antibody Alexa Fluor 594 (Thermo Fisher A-21203), Donkey anti-Rabbit IgG (H+L) secondary antibody Alexa Fluor 594 (Thermo Fisher A-21207), IRDye 680RD goat anti-Rabbit (Li-Cor 926-68071), IRDye 800CW goat anti-Mouse (Li-Cor 926-32210), IRDye 800CW donkey anti-Goat (Li-Cor 925-68074)

Validation

All antibodies are from commercially available and have been used extensively. Below are links to these products.
https://www.abcam.com/calnexin-antibody-er-marker-ab22595.html
https://www.abcam.com/ctip2-antibody-25b6-chip-grade-ab18465.html
https://www.abcam.com/ctip2-antibody-ab70453.html
https://www.abcam.com/darpp32-antibody-ep721y-ab40802.html
https://www.abcam.com/dlx1-antibody-ab167575.html
https://www.abcam.com/foxg1-antibody-chip-grade-ab18259.html
https://www.abcam.com/foxp1-antibody-jc12-ab32010.html
https://www.sigmaaldrich.com/catalog/product/sigma/a2052?lang=en&region=GB
https://www.abcam.com/gapdh-antibody-loading-control-ab9485.html
https://www.antibodies.com/gbx2-antibody-a84236
https://www.bdbiosciences.com/us/applications/research/intracellular-flow/intracellular-antibodies-and-isotype-controls/anti-rat-antibodies/purified-mouse-anti-ki-67-b56/p/550609
https://www.sigmaaldrich.com/catalog/product/sigma/m1406?lang=en&region=GB
https://www.merckmillipore.com/GB/en/product/Anti-Microtubule-Associated-Protein-2-MAP2-Antibody,MM_NF-AB5622
https://www.cellsignal.co.uk/products/primary-antibodies/nanog-d73g4-xp-rabbit-mab/4903
https://www.merckmillipore.com/GB/en/product/Anti-NeuN-Antibody-rabbit,MM_NF-ABN78
https://www.abcam.com/ttf1-antibody-ep1584y-ab76013.html
https://www.cellsignal.co.uk/products/primary-antibodies/oct-4a-c30a3-rabbit-mab/2840?_=1591442447814&Ntt=2840&tahead=true
https://www.rndsystems.com/products/human-mouse-olig3-antibody-257934_mab2456#product-details
https://www.abcam.com/pax6-antibody-epr15858-ab195045.html
https://www.abcam.com/psd95-antibody-synaptic-marker-ab18258.html
https://www.abcam.com/satb2-antibody-satba4b10-c-terminal-ab51502.html
https://www.cellsignal.co.uk/products/primary-antibodies/sox2-d6d9-xp-rabbit-mab/3579?_=1591442506226&Ntt=3579&tahead=true
https://www.scbt.com/p/sox-2-antibody-y-17
https://www.abcam.com/tbr1-antibody-ab31940.html
https://www.sigmaaldrich.com/catalog/product/sigma/t8660?lang=en&region=GB
https://www.thermofisher.com/antibody/product/Donkey-anti-Rabbit-IgG-H-L-Highly-Cross-Adsorbed-Secondary-Antibody-Polyclonal/A-21206
https://www.thermofisher.com/antibody/product/Donkey-anti-Rat-IgG-H-L-Highly-Cross-Adsorbed-Secondary-Antibody-Polyclonal/A-21208
https://www.thermofisher.com/antibody/product/Donkey-anti-Mouse-IgG-H-L-Highly-Cross-Adsorbed-Secondary-Antibody-Polyclonal/A-21203
https://www.thermofisher.com/antibody/product/Donkey-anti-Rabbit-IgG-H-L-Highly-Cross-Adsorbed-Secondary-Antibody-Polyclonal/A-21207
https://www.licor.com/bio/reagents/irdye-680rd-goat-anti-rabbit-igg-secondary-antibody

https://www.licor.com/bio/reagents/irdye-800cw-goat-anti-mouse-igg-secondary-antibody
https://www.licor.com/bio/reagents/irdye-680rd-donkey-anti-goat-igg-secondary-antibody

Eukaryotic cell lines

Policy information about cell lines

Cell line source(s)	H7 human embryonic stem cell line (Wi Cell, WA07 or H7); JSD4 DLG2+/+ hESC line (WT, this study); JSD2 DLG2-/- hESC line (KO1, this study); JSD21 DLG2-/- hESC line (KO2, this study)
Authentication	Pluripotency gene expression via immunocytochemistry, CNV analysis and quantification of DLG2 mRNA and protein by LC-MS/MS confirms the all three newly generated lines do not have additional CNVs in comparison to the parental H7 line, expresses pluripotency markers and lacks DLG2 protein in peptide-affinity pulldowns from KO lines.
Mycoplasma contamination	All cell lines were tested negative for mycoplasma contamination.
Commonly misidentified lines (See ICLAC register)	No commonly misidentified cell lines were used in the study.

Animals and other organisms

Policy information about studies involving animals; ARRIVE guidelines recommended for reporting animal research

Laboratory animals	Postnatal day 7-10 old Sprague-Dawley male and female rats (Charles River) were used to generate primary astrocytes to support human neurons in culture for electrophysiological recordings.
Wild animals	The study did not involve wild animals.
Field-collected samples	The study did not involve samples collected from the field.
Ethics oversight	All animal procedures were performed in accordance with Cardiff University's animal care committee's regulations and the European Directive 2010/63/EU on the protection of animals used for scientific purposes.

Note that full information on the approval of the study protocol must also be provided in the manuscript.
